# Nausea and vomiting as adverse events of oliceridine: a systematic review and meta-analysis of randomized controlled trials

**DOI:** 10.3389/fphar.2026.1779641

**Published:** 2026-04-22

**Authors:** Jinfang Zeng, Shu Yang, Jinjin Jian, Minmin Zhu

**Affiliations:** 1 Department of Anesthesiology and Pain Medicine, Wuxi No.2 People’s Hospital (Jiangnan University Medical Center), Wuxi, China; 2 Wuxi School of Medicine, Jiangnan University, Wuxi, China; 3 Department of Anesthesiology, Affiliated Hospital of Jiangnan University, Wuxi, China

**Keywords:** meta-analysis, nausea, oliceridine, TRV130, vomiting, PONV

## Abstract

**Background:**

Postoperative nausea and vomiting (PONV) continue to be some of the most common and troublesome complications following anesthesia. Oliceridine, a G protein-biased µ-opioid receptor agonist, has the potential to provide effective pain relief while reducing the incidence of opioid-associated side effects.

**Objectives:**

The aim of this study is to evaluate the effectiveness and safety of oliceridine for preventing PONV compared with morphine and a placebo.

**Methods:**

A comprehensive search was performed across PubMed/MEDLINE, Embase (Ovid), and the Cochrane Central Register of Controlled Trials (CENTRAL) from their inception up to 30 September 2025, without any language limitations. Randomized controlled trials (RCTs) comparing oliceridine with morphine or placebo were included. Outcomes included incidence of nausea, vomiting, and other opioid-related adverse events (ORAEs). Pooled risk ratios (RRs) and their corresponding 95% confidence intervals (CIs) were estimated using random-effects models.

**Results:**

This meta-analysis included five studies, with a total of 1,767 patients. The pooled analysis showed that oliceridine significantly reduced the incidence of postoperative nausea compared with morphine (risk ratio [RR] = 0.80; 95% confidence interval [CI] = 0.70–0.90). In dose-specific analyses, the RRs were 0.58 (95% CI = 0.50–0.67) for the 0.1 mg group, 0.82 (95% CI = 0.74–0.92) for the 0.35 mg group, and 1.00 (95% CI = 0.91–1.11) for the 0.5 mg group. Oliceridine also decreased the risk of postoperative vomiting (RR = 0.55; 95% CI = 0.45–0.67) relative to morphine. Subgroup analyses yielded RRs of 0.39 (95% CI = 0.31–0.50), 0.54 (95% CI = 0.38–0.78), and 0.80 (95% CI = 0.68–0.95) for the 0.1 mg, 0.35 mg, and 0.5 mg groups, respectively. Moreover, oliceridine appeared to lower the incidence of opioid-related adverse events, including dizziness (RR = 0.89; 95% CI = 0.76–1.04), dry mouth (RR = 0.50; 95% CI = 0.37–0.69), pruritus (RR = 0.50; 95% CI = 0.35–0.70), and somnolence (RR = 0.61; 95% CI = 0.45–0.82). Based on the Grading of Recommendations Assessment, Development and Evaluation (GRADE) system, the certainty of evidence ranged from moderate to low.

**Conclusion:**

Oliceridine, particularly at 0.1–0.35 mg demand doses, reduces PONV and several opioid-related adverse events compared with morphine while maintaining effective analgesia. However, compared with placebo, typical opioid adverse effects remain. Further large-scale RCTs are warranted.

**Systematic Review Registration:**

https://www.crd.york.ac.uk/PROSPERO/view/CRD42024604703, identifier PROSPERO (CRD42024604703).

## 1 Introduction

It is well recognized that postoperative nausea and vomiting (PONV) are common complications after general anesthesia (Kim et al., 2017). Past studies have revealed a link between PONV and a higher rate of negative occurrences, including respiratory depression, hypotension, and hypothermia (Kienbaum et al., 2022). Such actions might worsen the situation and extend hospitalizations. Consequently, patients often dread experiencing nausea and vomiting, particularly post-surgery for laparoscopy, laparotomy, and strabismus (Huang et al., 2023). Furthermore, several independent predictors of PONV have been identified, including smoking history, patient age and sex, susceptibility to motion sickness, and prior episodes of PONV, each contributing to approximately a 20% increase in risk (Ishikawa et al., 2022). In addition, the incidence of PONV may also be influenced by factors related to anesthetic techniques, the type of anesthesia administered, and postoperative pain management strategies (Sch et al., 2023). Opioid consumption poses a significant risk for patients suffering from PONV. Nausea and vomiting caused by opioids are frequent side effects, with patients considering them among the most upsetting complications after surgery (Feng et al., 2024). However, oliceridine, a novel μ-opioid receptor ligand, preferentially activates the G-protein pathway associated with analgesia, rather than the β-arrestin pathway associated with opioid-related adverse effects, and it differs structurally from naturally occurring opiates and their semisynthetic derivatives (Shah et al., 2021; Simons et al., 2023). Several studies have suggested that oliceridine may be associated with a lower incidence of PONV than morphine (Beard et al., 2021; Singla et al., 2019). Nevertheless, the currently available evidence remains inconclusive. The majority of the published data are derived from individual randomized trials or exploratory analyses, and no quantitative synthesis has specifically evaluated the efficacy of oliceridine in preventing PONV. In addition, differences across studies in surgical populations, comparator groups, dosing regimens, and outcome reporting may limit the consistency and generalizability of existing findings. Therefore, we performed a systematic review and meta-analysis to quantitatively assess the efficacy of oliceridine in preventing PONV.

## 2 Methods

A comprehensive meta-analysis was carried out to evaluate the impact of oliceridine on PONV, in compliance with the guidelines specified in the PRISMA declaration. The study was registered in the PROSPERO database under the number CRD42024604703. As this research is a systematic review and meta-analysis based solely on previously published studies, no new human subjects were recruited; therefore, ethical approval and informed consent were not applicable. Considering the infrequent occurrence of vomiting without nausea and the comparable incidence rates between PONV and postoperative nausea (PON), PONV was regarded as a surrogate for PON when the latter was not explicitly reported in individual trials. For cases of PONV or PON, the severity of nausea was assessed. For evaluating antiemetic impacts, a conventional 24-h period was employed, and for extended or briefer durations, the one closest to the 24-h threshold was chosen. The evaluation of nausea was conducted using a categorical grading system, where 0 signifies none, 1 indicates mild, 2 represents moderate, and 3 denotes severe.

### 2.1 Search approach and eligibility standards

**FIGURE 1:**
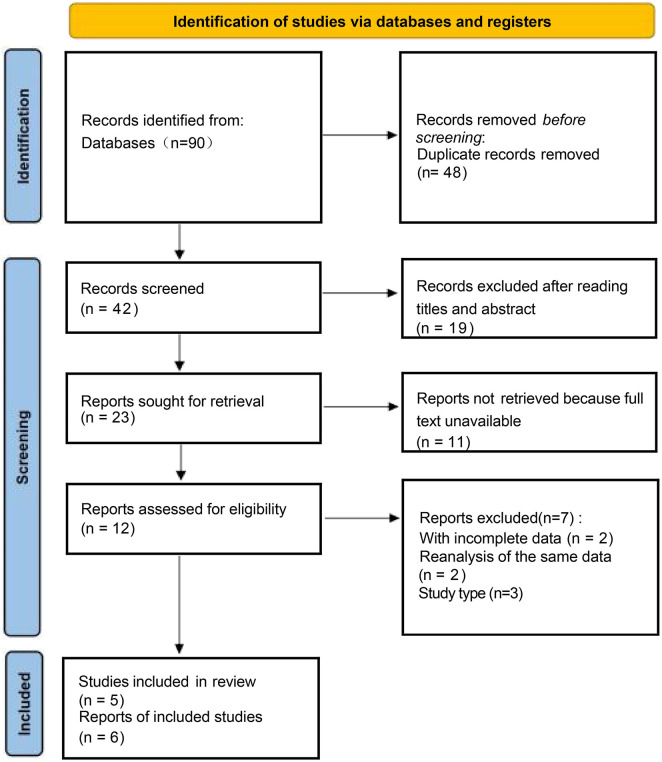
Flow diagram of the inclusion and exclusion processes.

A thorough literature search was independently conducted by two reviewers (Z.J.F. and J.J.J.) across PubMed/MEDLINE, Embase (Ovid), and the Cochrane Central Register of Controlled Trials (CENTRAL), covering all records available up to 30 September 2025. No restrictions were applied regarding language or publication status. Additionally, the bibliographies of relevant reviews and included studies were examined manually to identify any potentially eligible trials. The search strategy combined Medical Subject Headings (MeSH) and Emtree terms with free-text keywords related to oliceridine (TRV130), perioperative or postoperative contexts, and nausea or vomiting, along with filters specific to randomized controlled trials. All duplicate citations were removed before the screening process began. Titles/abstracts and then full texts were screened in duplicate against pre-specified eligibility criteria (adults ≥18 years; oliceridine used postoperatively or in volunteers; comparator morphine or placebo; randomized controlled design; outcomes including nausea or vomiting). Disagreements were resolved through discussion or by a third reviewer. The complete database-specific strategies and run dates are provided in the search strategy and study selection (Methods), and Supplementary Table S1; study selection is summarized in the PRISMA 2020 flow diagram (Figure 1).

### 2.2 Research selection

The extracted information included the first author’s name, year of publication, type and duration of anesthesia and surgery, intervention details, incidence of nausea and vomiting, and the total sample size. Two reviewers (Y.S. and Z.M.M.) independently evaluated the retrieved studies based on predefined inclusion and exclusion criteria. Any disagreements between the reviewers were resolved through discussion with the entire research team.

### 2.3 Inclusion criteria

Studies were included if they met all eligibility criteria, which were defined as follows: (1) population: adult patients (18 years and older); (2) intervention: oliceridine used postoperatively or in healthy volunteers; (3) comparator: placebo versus morphine; (4) primary outcomes: frequency of nausea or vomiting; secondary outcomes: dizziness, dry mouth, headache, hypotension, pruritus, somnolence, and surgery duration; and (5) types of studies: randomized controlled trials (RCTs).

### 2.4 Exclusion criteria

The exclusion criteria were as follows:

1. Studies available only as registration records or abstracts
2. Studies lacking essential outcome data
3. Studies with inappropriate or inaccurate statistical analyses.

### 2.5 Information extraction and evaluation of the bias risk

Two investigators (Z.M.M. and J.J.J.) independently assessed the methodological quality of the included studies using the Cochrane Collaboration’s Risk of Bias tool (ROB1). The following domains were evaluated: random sequence generation, allocation concealment, blinding of participants and personnel, blinding of outcome assessment, incomplete outcome data, selective reporting, and other bias. Each domain was judged as having low risk, unclear risk, or high risk of bias.

### 2.6 Quality analysis of evidence

The certainty of evidence for each outcome was assessed using the Grading of Recommendations Assessment, Development and Evaluation (GRADE) approach. Five domains were considered: risk of bias, inconsistency, indirectness, imprecision, and publication bias. The overall certainty of evidence was rated as high, moderate, low, or very low.

### 2.7 Trial sequential analysis

Trial sequential analysis (TSA) was performed using Trial Sequential Analysis software, version 0.9.5.10 beta [computer program]. The adequacy of the total sample size was assessed, and TSA was applied to evaluate whether further studies were required. The required information size was calculated while accounting for between-study heterogeneity. Diversity, indicative of the variability rate across trials, is determined by totaling the disparities among trials, factoring in the anticipated sampling error due to the necessary information size. TSA was performed with a predefined type I error rate of 5%, consistent with standards commonly applied in meta-analyses and systematic reviews. The required information size was calculated based on an α error of 5% and a β error of 20%. Convincing evidence was considered to be achieved when the cumulative Z-curve crossed the trial sequential monitoring boundary before reaching the required information size. Conversely, if the boundary was not crossed, further trials were deemed necessary to obtain conclusive results.

### 2.8 Outcome measures

The overall effect was evaluated using a Z-test, and statistical significance was defined as a *p*-value less than 0.05. This research utilized a framework grounded in stochastic influences. To assess oliceridine’s effectiveness against nausea and vomiting and the probability of dizziness, dry mouth, headache, hypotension, pruritus, and somnolence, the combined risk ratio (RR) was computed. To assess the surgery duration, the combined standardized mean difference (SMD) was employed, accompanied by a 95% confidence interval (CI). Subgroup analyses took into account variables such as the amount of oliceridine used.

Subgroup analyses: Pre-specified subgroup analyses were conducted to explore heterogeneity. For the primary outcomes (nausea and vomiting), we analyzed subgroups based on oliceridine demand dose (0.1 mg, 0.35 mg, and 0.5 mg) and comparator (morphine vs. placebo). Secondary outcomes (dizziness, dry mouth, headache, hypotension, pruritus, and somnolence) were similarly examined by dose and comparator, where data permitted. Pooled effects were calculated using random-effects models. Heterogeneity within each subgroup was assessed using *I* ^*2*^ and *χ* ^*2*^ (Cochran’s Q). Between-subgroup differences were evaluated using a test for subgroup differences (Q-test for interaction); a two-sided *p* < 0.10 was considered suggestive of an interaction. Where *I* ^*2*^ > 50% in a subgroup, we performed leave-one-out sensitivity analyses. Effect measures were RRs with 95% CIs for dichotomous outcomes and SMDs with 95% CIs for continuous outcomes.

Assessment of publication bias and small-study effects: Because few trials contributed to each outcome, funnel plots were constructed only where feasible. We used Begg’s and Egger’s tests and interpreted the results cautiously, given the low statistical power associated with a small number of studies. No formal sensitivity analyses (e.g., leave-one-out) were undertaken.

## 3 Results

### 3.1 Study selection

As depicted in the flowchart (Figure 1), delving into PubMed, Embase, the Cochrane Library, and bibliographies led to the uncovering of 90 scholarly articles. Initially, 48 trials were omitted because of their repetitive characteristics. Following an examination of titles and abstracts, 19 studies were excluded. Additionally, 11 experiments were excluded owing to the unavailability of the entire text. An in-depth examination of 12 studies revealed a lack of associated endpoints in 7, resulting in their exclusion. Ultimately, the meta-analysis included five studies (Singla et al., 2019; Hammer et al., 2021; Singla et al., 2017; Soergel et al., 2014; Viscusi et al., 2019) that met the criteria for selection.

### 3.2 Study characteristics

Among the studies considered, five trials (Singla et al., 2019; Hammer et al., 2021; Singla et al., 2017; Soergel et al., 2014; Viscusi et al., 2019) investigated oliceridine’s effectiveness against PONV (Table 1). All of the included studies were published in 2014 or later, and all of the enrolled participants were adults. The majority of the studies were Phase III trials, and the majority of participants were White. The disease type was abdominoplasty or bunionectomy. The control group was given morphine or a placebo.

**TABLE 1:** General information on patients with incidence of postoperative nausea and vomiting.

Author	Year	Phase	Ethnicity, n (%)	Age*continuous mean (SD) (years)	Sex, male subjects/female subjects)	BMI, kg m^-2^	Disease type	Comparisons (group)	PON (nausea)	POV (vomiting)	Total (case)
Hammer G. B.(a)	2021	Phase III	—	—	—	—	Abdominoplasty	Oliceridine 1.5/0.10 mg/mg	34	18	77
			—	—	—	—		Oliceridine 1.5/0.35 mg/mg	49	17	79
			—	—	—	—		Oliceridine 1.5/0.5 mg/mg	60	34	80
			—	—	—	—		Morphine 4.0/1.0 mg/mg	61	44	82
Hammer G. B.(b)	2021	Phase III	—	—	—	—	Bunionectomy	Oliceridine 1.5/0.10 mg/mg	27	13	76
			-	-	—	—		Oliceridine 1.5/0.35 mg/mg	44	31	79
			—	—	—	—		Oliceridine 1.5/0.5 mg/mg	50	32	79
			—	—	—	—		Morphine 4.0/1.0 mg/mg	49	38	76
Singla N.	2017	Phase IIb	White 107 (53.5%) Black/African American 82 (41%) Other 11 (5.5%)	37.2 ± 7.9	0/39	27.2 ± 3.1	Abdominoplasty	Oliceridine 1.5/0.10 mg/mg	16	6	39
				40.3 ± 12.0	0/39	26.1 ± 2.5		Oliceridine 1.5/0.35 mg/mg	18	6	39
				37.4 ± 9.2	2/37	26.5 ± 3.2		Placebo	7	3	39
				37.5 ± 9.1	0/83	26.9 ± 3.1		Morphine 4.0/1.0 mg/mg	60	35	83
Singla N. K.	2019	Phase III	White 270 (69.4%) Black/African American 94 (24.2%) Other 25 (6.4%)	41.8 ± 10.6	1/76	28.0 ± 3.4	Abdominoplasty	Oliceridine 1.5/0.10 mg/mg	34	18	77
				42.0 ± 10.0	0/80	27.6 ± 3.0		Oliceridine 1.5/0.35 mg/mg	49	17	80
				40.4 ± 10.0	0/80	27.0 ± 3.2		Oliceridine 1.5/0.5 mg/mg	60	34	80
				42.2 ± 10.3	0/81	27.0 ± 3.5		Placebo	38	11	81
				40.4 ± 10.4	2/81	26.8 ± 3.3		Morphine 4.0/1.0 mg/mg	61	44	83
Soergel D. G.	2014	—	​	—	29/0	—	Healthy volunteers	Oliceridine 1.5 mg	1	0	29
			​	—	30/0	—		Oliceridine 3 mg	7	1	30
			​	—	30/0	—		Oliceridine 4.5 mg	10	4	30
			​	—	30/0	​		Placebo	1	0	30
			​	—	30/0	—		Morphine 4.0/1.0 mg/mg	4	6	30
Viscusi E. R.	2019	Phase III	White 270 (69.4%) Black/African American 94 (24.2%) Other 25 (6.4%)	47.5 ± 12.7	12/64	26.4 ± 4.4	Bunionectomy	Oliceridine 1.5/0.10 mg/mg	27	13	76
				43.6 ± 13.9	14/65	26.0 ± 3.8		Oliceridine 1.5/0.35 mg/mg	44	31	79
				46.9 ± 13.8	13/66	27.1 ± 4.3		Oliceridine 1.5/0.5 mg/mg	50	32	79
				44.1 ± 12.6	9/70	26.3 ± 4.3		Placebo	19	5	79
				43.3 ± 14.1	11/65	26.5 ± 4.5		Morphine 4.0/1.0 mg/mg	49	38	76

Abbreviations: PON, postoperative nausea; POV, postoperative vomiting; PONV, postoperative nausea and vomiting; BMI, body mass index; SD, standard deviation; mg, milligram; kg, kilogram; m^2^, square meter.

### 3.3 Methodological quality of the included studies

**FIGURE 2:**
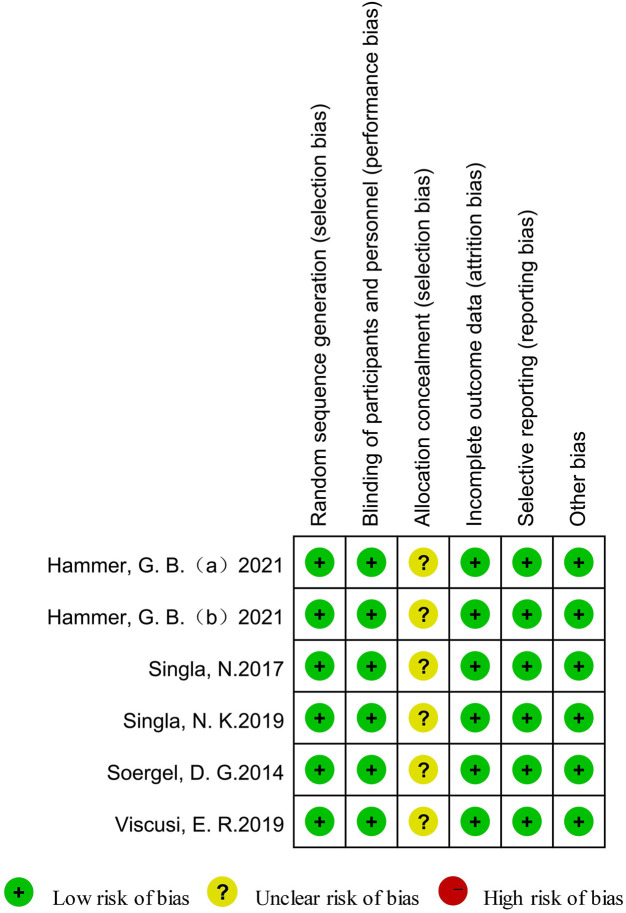
Summary of the risk of bias of the included studies.

Overall, the included studies were not judged to be at high risk of bias across the majority of domains. Two studies (Singla et al., 2019; Soergel et al., 2014) reported using random number tables, and two studies (Singla et al., 2017; Viscusi et al., 2019) adopted computer-generated random numbers. One study (Hammer et al., 2021) reported the use of sealed envelopes; however, insufficient details were provided to fully judge the allocation concealment process. Overall, all trials lacked sufficient information regarding allocation concealment, and this domain was, therefore, rated as having an unclear risk of bias. Because unclear allocation concealment may introduce potential selection bias, the methodological quality of the included studies and the pooled findings should be interpreted with caution. All of the included trials (Singla et al., 2019; Hammer et al., 2021; Singla et al., 2017; Soergel et al., 2014; Viscusi et al., 2019) were completed without any participant withdrawals, and all predefined outcomes described in the *Methods* section were reported. Figure 2 presents the detailed risk-of-bias assessment.

### 3.4 Quality of evidence

GRADE assessments of the certainty of evidence showed that I^2^ values indicated moderate or high thresholds for statistical heterogeneity. There were studies sponsored by pharmaceutical companies; the total number of events was less than 300, and RR < 0.2. Every study utilized randomized approaches, with the effectiveness of oliceridine in treating nausea assessed as low-certainty evidence, while its impact on vomiting was considered moderate-certainty evidence (Table 2).

**TABLE 2:** GRADE summary of the efficacy of oliceridine

Quality assessment							No. of patients		Effect		Quality	Importance
No. of studies	Design	Risk of bias	Inconsistency	Indirectness	Imprecision	Other considerations	Oliceridine	Control	Relative (95% CI)	Absolute
Nausea												
5	Randomized trials	Serious^a^	Serious^b^	No serious indirectness	No serious imprecision	Reporting bias^c^	204/356 (57.3%)	280/400 (70%)	RR 0.82 (0.74–0.92)	126 fewer per 1,000 (95% CI 56 to 182 fewer)	⊕⊕ΟΟ LOW	CRITICAL
							​	72.3%		130 fewer per 1,000 (95% CI 58 to 188 fewer)		
Vomiting												
5	Randomized trials	Serious^a^	No serious inconsistency	No serious indirectness	No serious imprecision	Reporting bias^c^	102/356 (28.7%)	199/400 (49.8%)	RR 0.54 (0.38–0.78)	229 fewer per 1,000 (95% CI 109 to 308 fewer)	⊕⊕⊕Ο MODERATE	CRITICAL
							​	50%		230 fewer per 1000 (95% CI 110 to 310 fewer)		
Headache												
3	Randomized trials	No serious risk of bias	Serious^b^	No serious indirectness	Serious^d^	None	49/198 (24.7%)	61/242 (25.2%)	RR 0.92 (0.66–1.27)	20 fewer per 1,000 (95% CI 86 to 68 more)	⊕⊕ΟΟ LOW	IMPORTANT
							​	28.9%		23 fewer per 1000 (95% CI 98 to 78 more)		
​	​	​	​	​	​	​	​	6.7%	​	0 fewer per 1000 (95% CI 57 to 378 more)	​	​
Dizziness												
5	Randomized trials	No serious risk of bias	Serious^b^	No serious indirectness	Serious^d^	Reporting bias^c^	68/356 (19.1%)	85/400 (21.3%)	RR 0.83 (0.63–1.09)	36 fewer per 1,000 (95% CI 79 to 19 more)	⊕ΟΟΟ VERY LOW	IMPORTANT
							​	15.9%		27 fewer per 1,000 (95% CI 59 to 14 more)		
Somnolence												
5	Randomized trials	No serious risk of bias	No serious inconsistency	No serious indirectness	Serious^d^	Reporting bias^c^	62/356 (17.4%)	84/400 (21%)	RR 0.77 (0.42–1.44)	48 fewer per 1,000 (95% CI 122 to 92 more)	⊕⊕ΟΟ LOW	IMPORTANT
							​	15.8%		36 fewer per 1,000 (95% CI 92 to 70 more)		
							​	26.7%		32 more per 1,000 (95% CI 134 to 406 more) —		
Hypotension												
1	Randomized trials	No serious risk of bias	Serious^b^	No serious indirectness	Serious^d^	None	3/39 (7.7%)	7/83 (8.4%)	RR 0.91 (0.25–3.34)	8 fewer per 1,000 (95% CI 63 to 197 more)	⊕⊕ΟΟ LOW	IMPORTANT
							​	8.4%		8 fewer per 1,000 (95% CI 63 to 197 more)		
Pruritus												
4	Randomized trials	No serious risk of bias	Serious^b^	No serious indirectness	Serious^d^	None	54/317 (17%)	86/317 (27.1%)	RR 0.63 (0.46–0.85)	100 fewer per 1,000 (95% CI 41 to 146 fewer)	⊕⊕ΟΟ LOW	IMPORTANT
							​	27.4%		101 fewer per 1,000 (95% CI 41 to 148 fewer)		
Dry mouth												
2	Randomized trials	No serious risk of bias	No serious inconsistency	No serious indirectness	Serious^d^	Reporting bias^b^	20/159 (12.6%)	31/159 (19.5%)	RR 0.65 (0.39–1.09)	68 fewer per 1,000 (95% CI 119 to 18 more)	⊕⊕ΟΟ LOW	IMPORTANT
							​	19.3%		68 fewer per 1,000 (95% CI 118 to 17 more)		
​	​	​	​	​	​	​	​	10%	​	0 fewer per 1,000 (95% CI 78 to 356 more)	​	​
Surgery duration												
2	Randomized trials	No serious risk of bias	Serious^b^	No serious indirectness	Serious^e^	Very strong association^f^	159	159	—	SMD 0.09 higher (0.13 lower to 0.31 higher)	⊕⊕⊕⊕ HIGH	IMPORTANT

^a^ Downgraded due to unclear allocation concealment across included trials.

^b^ I^2^ values indicated moderate or high thresholds for statistical heterogeneity.

^c^ Studies sponsored by pharmaceutical companies.

^d^ Total number of events is less than 300.

^e^ Total population size is less than 400.

^f^ RR<0.2.

Abbreviations: RR, risk ratio; CI, confidence interval; SMD, standardized mean difference; I^2^, inconsistency index; GRADE, Grading of Recommendations Assessment, Development and Evaluation; RCT, randomized controlled trial.

### 3.5 Outcomes versus morphine

All of the effects below compare oliceridine with morphine, and are subgrouped by oliceridine demand dose (0.1, 0.35, and 0.5 mg).

#### 3.5.1 Nausea—subgroup analysis by dose

A random-effects model and subgroup analyses were applied to evaluate heterogeneity in the comparison between oliceridine and morphine. The pooled estimate indicated that oliceridine significantly reduced the incidence of postoperative nausea compared with morphine (RR = 0.80; 95% CI = 0.70–0.90; *p* = 0.0004), with moderate heterogeneity observed among the included studies (I^2^ = 67%; *p* < 0.001).

**FIGURE 3:**
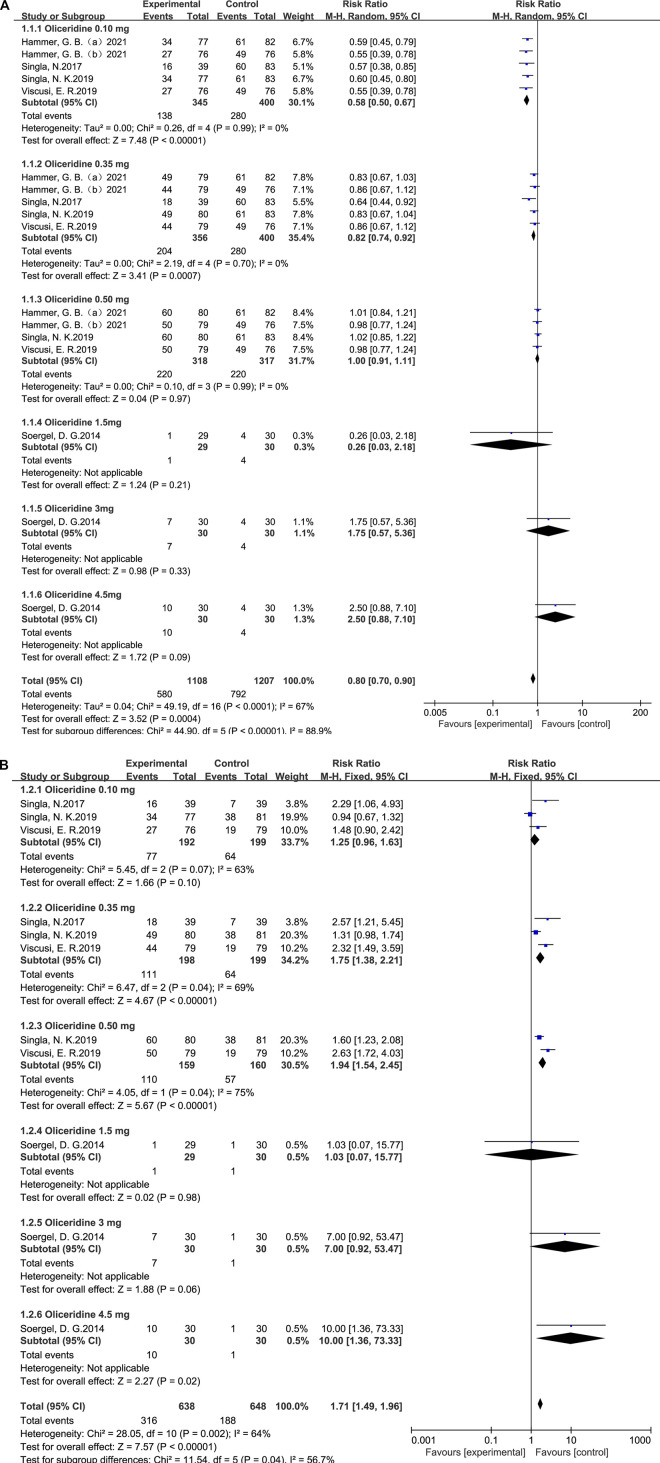
**(A)** Results of the incidence of postoperative nausea between oliceridine and morphine. **(B)** Results of the incidence of postoperative nausea between oliceridine and placebo.

In the subgroup analysis by dose, the relative risks were 0.58 (95% CI = 0.50–0.67; *p* < 0.001; I^2^ = 0%) for the 0.1 mg group, 0.82 (95% CI = 0.74–0.92; *p* = 0.0007; I^2^ = 0%) for the 0.35 mg group, and 1.00 (95% CI = 0.91–1.11; *p* = 0.97; I^2^ = 0%) for the 0.5 mg group (Figure 3A).

#### 3.5.2 Vomiting—subgroup analysis by dose

A random-effects model and subgroup analyses were used to assess heterogeneity in the comparison between oliceridine and morphine for postoperative vomiting. The pooled analysis demonstrated that oliceridine significantly reduced the incidence of vomiting compared with morphine (RR = 0.55; 95% CI = 0.45–0.67; *p* < 0.001), with moderate heterogeneity across the included studies (I^2^ = 62%; *p* = 0.0004).

**FIGURE 4:**
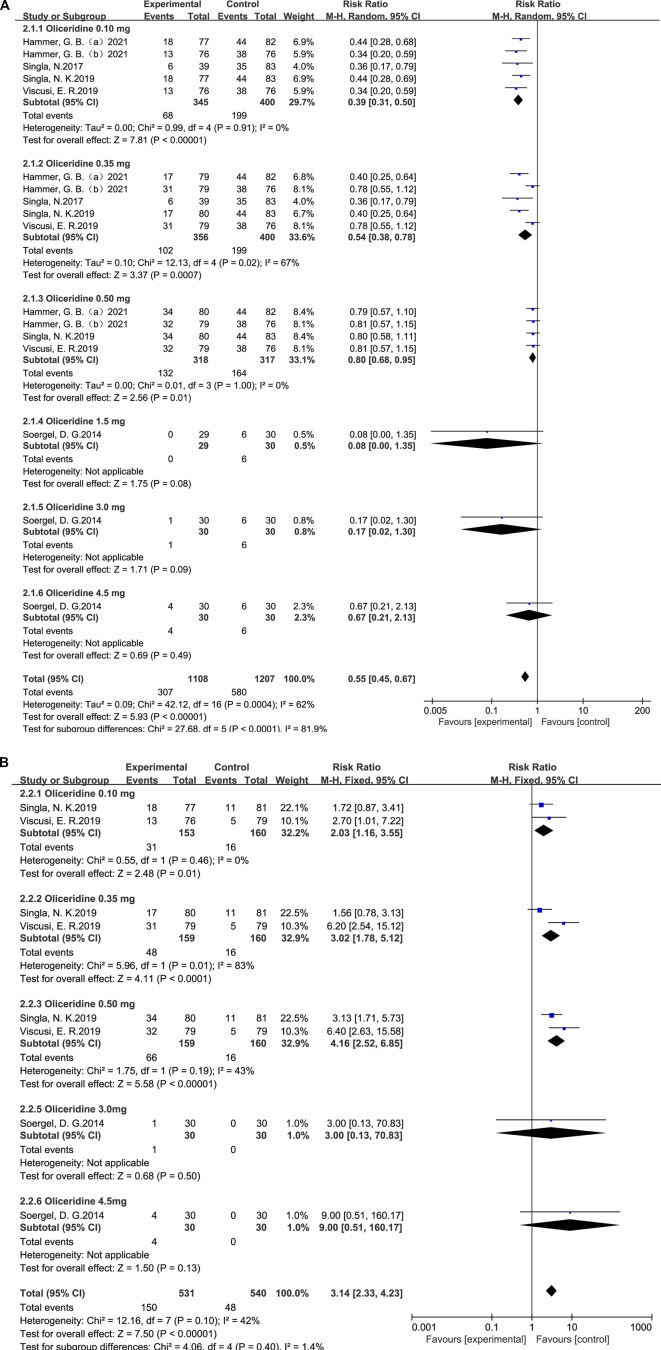
**(A)** Results of the incidence of postoperative vomiting between oliceridine and morphine. **(B)** Results of the incidence of postoperative vomiting between oliceridine and placebo.

In the dose-based subgroup analysis, the relative risks were 0.39 (95% CI = 0.31–0.50; *p* < 0.001; I^2^ = 0%) for the 0.1 mg group, 0.54 (95% CI = 0.38–0.78; *p* = 0.0007; I^2^ = 67%) for the 0.35 mg group, and 0.80 (95% CI = 0.68–0.95; *p* = 0.01; I^2^ = 0%) for the 0.5 mg group (Figure 4A).

#### 3.5.3 Secondary outcomes

##### 3.5.3.1 Dizziness—subgroup analysis by dose

**FIGURE 5:**
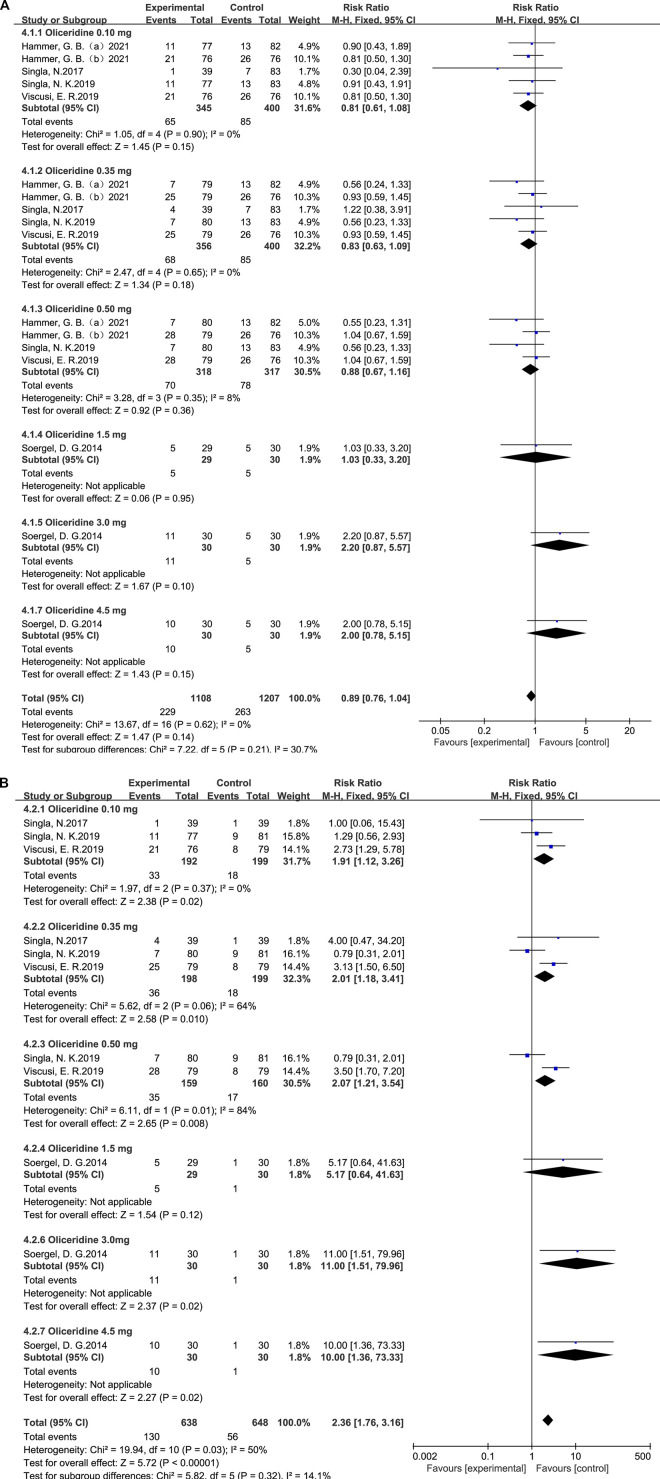
**(A)** Results of the probability of dizziness between oliceridine and morphine. **(B)** Results of the probability of dizziness between oliceridine and placebo.

Patients taking oliceridine were compared with those receiving morphine treatment for subgroup analysis. A baseline change in dizziness for oliceridine compared to morphine was observed (RR = 0.89; 95% CI = 0.76–1.04; *p* = 0.14), and the combined studies were heterogeneous (*p* = 0.62; I^2^ = 0%) (Figure 5A).

##### 3.5.3.2 Dry mouth—subgroup analysis by dose

Dry mouth associated with oliceridine compared to morphine was observed (RR = 0.50; 95% CI = 0.37–0.69; *p* < 0.001), and the combined studies were heterogeneous (*p* = 0.16; I^2^ = 32%) (Supplementary Figure S1A).

##### 3.5.3.3 Headache—subgroup analysis by dose

Headache associated with oliceridine compared to morphine was observed (RR = 0.91; 95% CI = 0.75–1.10; *p* = 0.32), and the combined studies were heterogeneous (*p* = 0.65; I^2^ = 0%) (Supplementary Figure S2A).

##### 3.5.3.4 Hypotension—subgroup analysis by dose

Hypotension associated with oliceridine compared to morphine was observed (RR = 1.37; 95% CI = 0.62–3.02; *p* = 0.44), and the combined studies were heterogeneous (*p* = 0.41; I^2^ = 0%) Supplementary Figure S3A).

##### 3.5.3.5 Pruritus—subgroup analysis by dose

Pruritus associated with oliceridine compared to morphine was observed (RR = 0.50; 95% CI = 0.35–0.70; *p* < 0.001), and the combined studies were heterogeneous (p = 0.002; I^2^ = 59%) (Supplementary Figure S4A).

##### 3.5.3.6 Somnolence—subgroup analysis by dose

Somnolence associated with oliceridine compared to morphine was observed (RR = 0.61; 95% CI = 0.45–0.82; *p* = 0.001), and the combined studies were heterogeneous (*p* = 0.004; I^2^ = 55%) (Supplementary Figure S5A).

##### 3.5.3.7 Surgery duration—subgroup analysis by dose

Surgery duration associated with oliceridine compared to morphine was observed (SMD = −0.02; 95% CI = −0.15 to 0.11; *p* = 0.78), and the combined studies were heterogeneous (*p* = 0.65; I^2^ = 0%) (Supplementary Figure S6A).

### 3.6 Outcomes versus placebo

All of the effects below compare oliceridine with placebo, and are subgrouped by oliceridine demand dose (0.1, 0.35, and 0.5 mg).

#### 3.6.1 Nausea—subgroup analysis by dose

In contrast to the placebo, a random-effects model and subgroup analysis were used to examine the variability in outcomes. The patient response rate for oliceridine for nausea, compared with placebo (RR = 1.71, 95% CI = 1.49–1.96, *p* < 0.001), was heterogeneous across the combined studies (*p* = 0.002, I^2^ = 64%); we performed a subgroup analysis based on the dose of oliceridine, compared with placebo: 0.1 mg dose group (RR = 1.25; 95% CI = 0.96–1.63; *p* = 0.10; I^2^ = 63%), 0.35 mg dose group (RR = 1.75; 95% CI = 1.38–2.21; *p* < 0.001; I^2^ = 69%), and 0.5 mg dose group (RR = 1.94; 95% CI = 1.54–2.45; *p* < 0.001; I^2^ = 75%) (Figure 3B).

#### 3.6.2 Vomiting—subgroup analysis by dose

Compared with placebo, a random-effects model and subgroup analyses were conducted to assess heterogeneity in the outcomes. The pooled analysis showed that oliceridine significantly increased the incidence of vomiting compared with placebo (RR = 3.14; 95% CI = 2.33–4.23; *p* < 0.001), with moderate heterogeneity among the included studies (I^2^ = 42%; *p* = 0.10).

Subgroup analysis by oliceridine dose demonstrated relative risks of 2.03 (95% CI = 1.16–3.55; *p* = 0.01; I^2^ = 0%) for the 0.1 mg group, 3.02 (95% CI = 1.78–5.12; *p* < 0.001; I^2^ = 83%) for the 0.35 mg group, and 4.16 (95% CI = 2.52–6.85; *p* < 0.001; I^2^ = 43%) for the 0.5 mg group (Figure 4B).

#### 3.6.3 Secondary outcomes

##### 3.6.3.1 Dizziness—subgroup analysis by dose

A baseline change was observed in oliceridine compared to placebo (RR = 2.36; 95% CI = 1.76–3.16; *p* < 0.001), and the combined studies were heterogeneous (p = 0.03; I^2^ = 50%) (Figure 5B).

##### 3.6.3.2 Dry mouth—subgroup analysis by dose

Dry mouth associated with oliceridine was observed compared to placebo (RR = 3.24; 95% CI = 1.88–5.6; *p* < 0.001), and the combined studies were homogeneous (*p* = 0.92; I^2^ = 0%) (Supplementary Figure S1B).

##### 3.6.3.3 Headache—subgroup analysis by dose

Headache associated with oliceridine was observed compared to placebo (RR = 0.90; 95% CI = 0.74–1.09; *p* = 0.27), and the combined studies were homogeneous (*p* = 0.61; I^2^ = 0%) (Supplementary Figure S2B).

##### 3.6.3.4 Hypotension—subgroup analysis by dose

Hypotension associated with oliceridine was observed compared to placebo (RR = 4.5; 95% CI = 1.00–20.16; *p* = 0.05), and the combined studies were homogeneous (*p* = 0.65; I^2^ = 0%) (Supplementary Figure S3B).

##### 3.6.3.5 Pruritus—subgroup analysis by dose

Pruritus associated with oliceridine was observed compared to placebo (RR = 2.00; 95% CI = 1.35–2.98; *p* = 0.0006), and the combined studies were homogeneous (*p* = 0.20; I^2^ = 29%) (Supplementary Figure S4B).

##### 3.6.3.6 Somnolence—subgroup analysis by dose

Somnolence associated with oliceridine compared to placebo was observed (RR = 2.01; 95% CI = 1.42–2.84; p < 0.001), and the combined studies were homogeneous (*p* = 0.26; I^2^ = 20%) (Supplementary Figure S5B).

##### 3.6.3.7 Surgery duration—subgroup analysis by dose

Surgery duration for oliceridine compared to placebo showed no significant difference (SMD = 0.02; 95% CI = −0.11 to 0.14; *p* = 0.80), and the combined studies were homogeneous (*p* = 0.78; I^2^ = 0%) (Supplementary Figure S6B).

Heterogeneity: Several pooled effects showed moderate to substantial heterogeneity (e.g., nausea vs. morphine I^2^ = 67%; vomiting vs. morphine I^2^ = 62%; nausea vs. placebo I^2^ = 64%; pruritus vs. morphine I^2^ = 59%), while others showed low or no heterogeneity. The pre-specified dose (0.1, 0.35, and 0.5 mg) and comparator (morphine vs. placebo) subgroups explained part of this variability, revealing dose-responsive patterns against placebo and greater benefits at lower doses versus morphine (Figures 3, 4, and Supplementary Figures).

### 3.7 Publication bias

**FIGURE 6:**
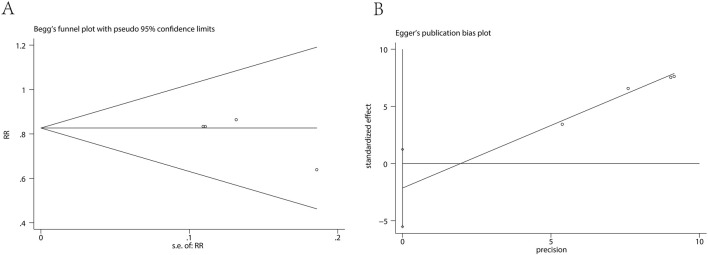
Publication bias assessment for nausea (oliceridine vs. morphine). **(A)** Begg’s funnel plot with pseudo 95% confidence limits. **(B)** Egger’s regression plot (standardized effect vs. precision).

Funnel-plot asymmetry was assessed using Begg’s and Egger’s methods where feasible (Figure 6). For the nausea comparison (oliceridine vs. morphine), Begg’s test (*p* = 0.807) and Egger’s test (*p* = 0.137) indicated no evidence of small-study effects. However, because few trials contributed to each outcome, the diagnostic power of these tests is limited, and inferences should be made cautiously.

### 3.8 Trial sequential analysis

TSA was applied to the nausea outcomes of five randomized controlled trials, comprising a total of 756 patients. The calculated sample size, adjusted for essential diversity, was determined to maintain a 5% risk of type I error and a 20% risk of type II error. The combined Z-curve transcended the limits of ineffectiveness and observation to reach a level of informational significance, signifying concrete evidence of oliceridine’s significant advantage over morphine in reducing nausea (Supplementary Figure S7A).

Furthermore, a TSA was conducted on vomiting cases, based on findings from five RCTs that included 756 patients. The aggregate Z-curve crossed the boundaries for both futility and monitoring before attaining the informational magnitude, signifying solid proof of oliceridine’s substantial superiority over the morphine group in alleviating vomiting (Supplementary Figure S7B).

## 4 Discussion

### 4.1 Overview

Post-anesthesia, PONV frequently emerged as a significant post-surgery issue, especially in the perioperative field (Yokoyama et al., 2020). Up to 30% of the total population experiences PONV (Scharfetter et al., 2024). Furthermore, PONV, a persistent issue, profoundly affects patients, disrupts the equilibrium of water and electrolytes, and in extreme scenarios, may result in aspiration pneumonitis, delayed hospital discharge, increased hospital expenses, and greater financial strain on patients (Kim et al., 2024; Ding et al., 2023). For patients, averting PONV is considered a primary objective in anesthesia (Kampo et al., 2019). The emergence of PONV is attributed to three distinct risk elements: each patient’s unique characteristics, the anesthesia technique employed, and surgical procedure-related factors (Zeng et al., 2024). Opioids are utilized during and post-surgery, presenting hazards linked to anesthesia methods. Despite extensive research in recent years, PONV continues to be a major obstacle, with the choice of anesthetic agents potentially affecting its occurrence (Horn et al., 2014). We, therefore, synthesized randomized evidence on oliceridine with dose-defined subgroups and comparator-specific results.

### 4.2 Comparison with morphine

Compared with morphine, oliceridine was associated with a lower incidence of nausea and vomiting, with the largest reductions at demand doses of 0.1–0.35 mg and a smaller or null effect at 0.5 mg (primary outcomes). For adverse events, oliceridine reduced dry mouth, pruritus, and somnolence, whereas dizziness, headache, hypotension, and surgery duration showed no material differences. These findings suggest that, at equianalgesic exposure, oliceridine may offer a more favorable PONV/ORAE profile than morphine, particularly at lower demand doses.

### 4.3 Comparison with placebo (blank control)

As expected for an active µ-opioid agonist, oliceridine showed higher rates of PONV and certain adverse events (e.g., dizziness, dry mouth, pruritus, and somnolence) than placebo, with a dose-responsive increase across 0.1–0.5 mg. These results contextualize the morphine comparison: although oliceridine improves tolerability compared to morphine, it still carries the typical opioid-related adverse effects relative to a blank control.

### 4.4 Publication bias and small-study effects

Visual inspection of the Begg’s funnel plot and Egger’s regression (Figure 6) did not suggest notable small-study effects for the nausea comparison (oliceridine vs. morphine); Begg’s and Egger’s tests were not significant. Nonetheless, because each outcome involved few trials, the statistical power of these analyses is limited, and selective reporting cannot be excluded.

### 4.5 Sources of heterogeneity

Between-study variability likely reflects differences in oliceridine dose demand (0.1–0.5 mg), comparator (morphine vs. placebo), procedure/anesthetic technique (e.g., abdominoplasty vs. bunionectomy), baseline PONV risk, antiemetic co-interventions, and assessment windows (time points near 24 h). Our pre-specified dose/comparator subgroups reduced inconsistency and yielded biologically plausible patterns (e.g., a greater benefit vs. morphine at lower doses; dose-responsive increases vs. placebo).

### 4.6 Mechanistic interpretation

Conventional opioids, such as morphine, alleviate pain by binding to μ-opioid receptors situated in the brain (Ramos-Gonzalez et al., 2023). This triggers a series of cellular activities, notably the activation of the G protein, which is believed to be the key facilitator of pain relief. Simultaneous enlistment of β-arrestin diminishes. The activation of G proteins is linked to an increase in negative incidents associated with opioids (Azzam et al., 2019). The μ-opioid receptor mediates both the pain relief and negative impacts of standard opioids, suggesting an unbreakable link between these effects. Conventional opioids amplify the binding of β-arrestin to the μ-opioid receptor, thereby obstructing the linkage of G proteins and consequently diminishing the effectiveness of pain relief (Al-Hasani and Bruchas, 2011). Investigating whether varied activation of μ-opioid pathways can dissociate analgesia from adverse effects, we identified oliceridine, a μ-opioid receptor ligand that favors “G protein” signaling with greater G protein-coupling efficacy compared to morphine, while exhibiting significantly reduced receptor phosphorylation, β-arrestin activation, and absorption (Markham, 2020).

Oliceridine, a new μ-opioid receptor ligand, serves as a full agonist to activate G proteins; however, it significantly lowers β-arrestin recruitment compared to traditional opioids, such as morphine or fentanyl (Dahan et al., 2020; Singleton et al., 2021). This substance favors the G-protein pathway over morphine and fentanyl, a preference nearly triple that of β-arrestin, and is termed a “biased ligand” (Moss et al., 2023; Mafi et al., 2020). In rodent studies, oliceridine demonstrates strong analgesic effects and is associated with reduced gastrointestinal side effects and respiratory depression compared to morphine when administered at equianalgesic doses (Litman, 2020). These results indicate that oliceridine may have a wider therapeutic index than morphine and suggest that oliceridine doses, associated with reduced negative reactions, can lead to effective pain alleviation (Simons et al., 2023). Consequently, the development of a ligand favoring G protein, such as oliceridine, which enhances pain relief and diminishes side effects reliant on β-arrestin, might mark a significant progression in opioid treatment (Viscusi et al., 2016).

Nausea and vomiting caused by opioids rank as some of the most frequent opioid-related adverse events (ORAEs), with patients considering these some of the most troubling post-surgical complications (Ni et al., 2024). Although not fatal, nausea and vomiting significantly distress patients. The results of our study reveal that oliceridine markedly lessens nausea and vomiting compared to morphine, which is consistent with earlier studies (Soergel et al., 2014; Nie et al., 2023; Daksla et al., 2023). Our findings indicate a dose-responsive yet reduced incidence of nausea and vomiting in patients undergoing oliceridine treatments as opposed to morphine. The most noticeable disparities were observed in oliceridine within the 0.1 mg and 0.35 mg dosage requirements. Oliceridine, a novel μ-opioid receptor ligand, tends to activate the pain-relieving G-protein route instead of the β-arrestin route, associated with opioid adverse effects (Yekkirala et al., 2019). The preference could be attributed to oliceridine’s role as a ligand that favors G-proteins on the μ-opioid receptor (Mafi et al., 2020). Furthermore, oliceridine, in contrast to morphine, lacks an identified active metabolite, possibly leading to more consistent results (Chen et al., 2013). Although opioids can cause nausea and vomiting as side effects, proper management of pain can also alleviate PONV. It is recognized that surgical trauma can cause post-surgical pain in patients, potentially leading to PONV among those under mental stress (Zeng et al., 2024). Neglecting proper pain control post-surgery can lead to patient discomfort, PONV, heightened sensitivity to pain, respiratory challenges, and a range of other problems. As a result, oliceridine could reduce PONV by alleviating postoperative pain. Our findings also indicate that oliceridine has the potential to reduce the incidence of dry mouth, pruritus, and somnolence.

### 4.7 Clinical implications

Acute pain guidelines recommend the lowest effective opioid dose to mitigate ORAEs (Khaw et al., 2021; Majid et al., 2023). When an opioid is required, and morphine would otherwise be used, 0.1–0.35 mg of oliceridine may balance analgesia and tolerability more favorably. In multimodal pathways prioritizing opioid sparing, the absolute risk of PONV with oliceridine vs. placebo should be weighed carefully.

### 4.8 Limitations and suggestions for practice

The certainty for several outcomes was low under GRADE due to the small number of studies and imprecision. The limited number of trials restricts firm conclusions, especially for dose–response comparisons and less common adverse events. Key PONV risk factors (e.g., history of motion sickness and non-smoking status) were inconsistently reported and could not be uniformly analyzed. A notable limitation of this meta-analysis is that allocation concealment was insufficiently reported in all included trials, which may have introduced potential selection bias and reduced confidence in the pooled estimates. In addition, because only a small number of trials were available for the majority of outcomes, the statistical power to detect publication bias was limited; therefore, publication bias and selective reporting cannot be fully excluded.

Additional adequately powered RCTs—stratified by baseline PONV risk and comparing equianalgesic regimens—are needed to confirm dose–response patterns and generalizability.

## 5 Conclusion and recommendations

In summary, oliceridine administration was associated with a lower incidence of PONV, along with less dry mouth, pruritus, and somnolence, compared with morphine administration. These findings suggest that oliceridine may offer certain advantages in postoperative recovery. In conclusion, current evidence suggests that oliceridine may have a more favorable adverse effect profile than morphine, especially regarding postoperative nausea and vomiting; however, further high-quality studies are needed to confirm these findings.

## Data Availability

The original contributions presented in the study are included in the article/Supplementary Material; further inquiries can be directed to the corresponding author.

## Supplementary material

SUPPLEMENTARY FIGURE 1 Results of the probability of dry mouth between oliceridine and morphine **(A)**. Results of the probability of dry mouth between oliceridine and placebo **(B)**.

SUPPLEMENTARY FIGURE 2 Results of the probability of headache between oliceridine and morphine **(A)**. Results of the probability of headache between oliceridine and placebo **(B)**.

SUPPLEMENTARY FIGURE 3 Results of the probability of hypotension between oliceridine and morphine **(A)**. Results of the probability of hypotension between oliceridine and placebo **(B)**.

SUPPLEMENTARY FIGURE 4 Results of the probability of pruritus between oliceridine and morphine **(A)**. Results of the probability of pruritus between oliceridine and placebo **(B)**.

SUPPLEMENTARY FIGURE 5 Results of the probability of somnolence between oliceridine and morphine **(A)**. Results of the probability of somnolence between oliceridine and placebo **(B)**.

SUPPLEMENTARY FIGURE 6 Results of surgery duration between oliceridine and morphine **(A)**. Results of surgery duration between oliceridine and placebo **(B)**.

SUPPLEMENTARY FIGURE 7 Trial sequential analysis results. **(A)** Trial sequential analysis for nausea. **(B)** Trial sequential analysis for vomiting.
