# Supplementary figures and images for: Nausea and vomiting as adverse events of oliceridine: a systematic review and meta-analysis of randomized controlled trials
Source: Front Pharmacol. 2026 Apr 22;17:1779641. doi: 10.3389/fphar.2026.1779641 (PMC13144020; doi:10.3389/fphar.2026.1779641)

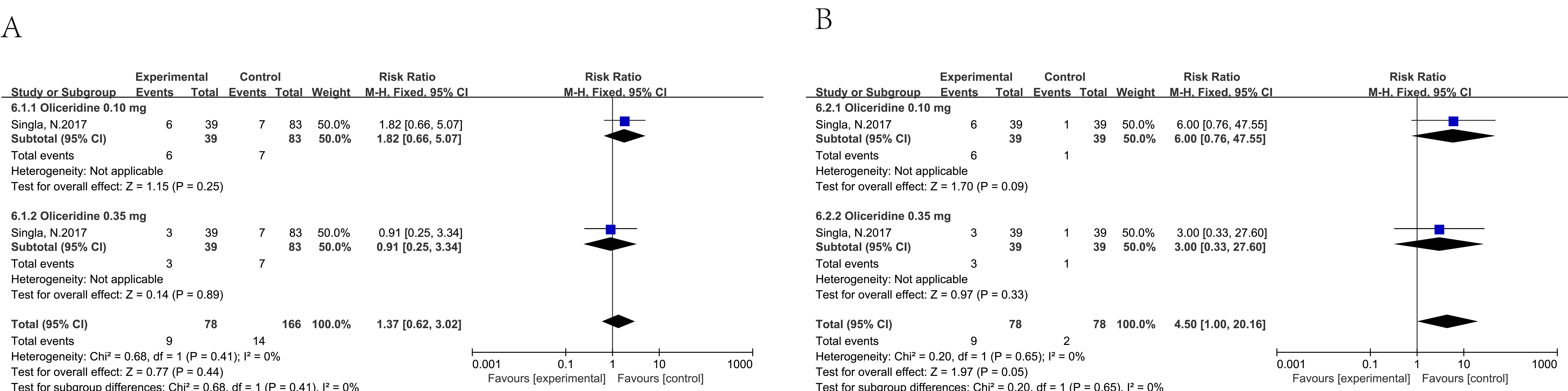

Supplement: Supplementary file 1 [file Image3.tiff]

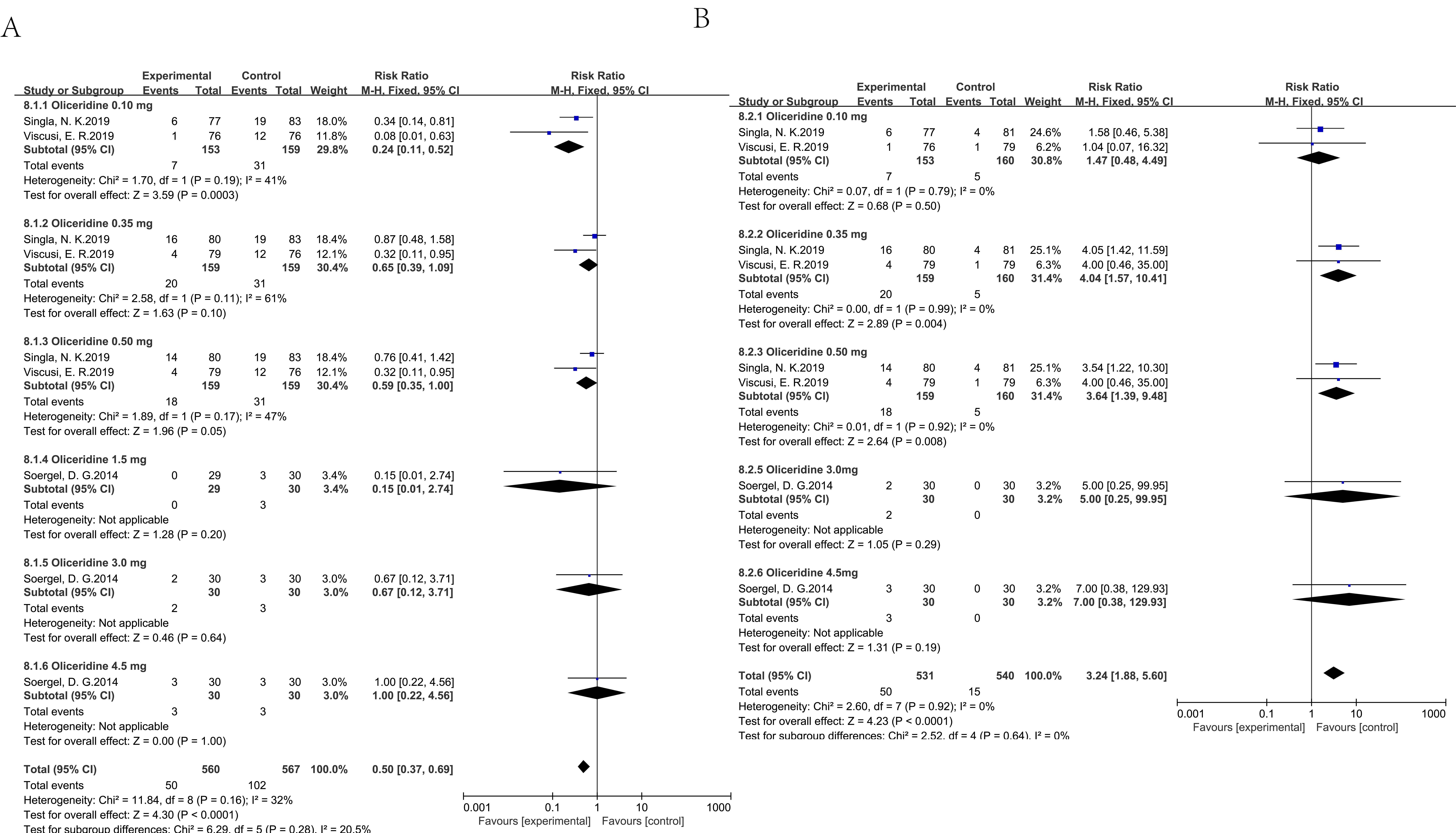

Supplement: Supplementary file 2 [file Image1.tiff]

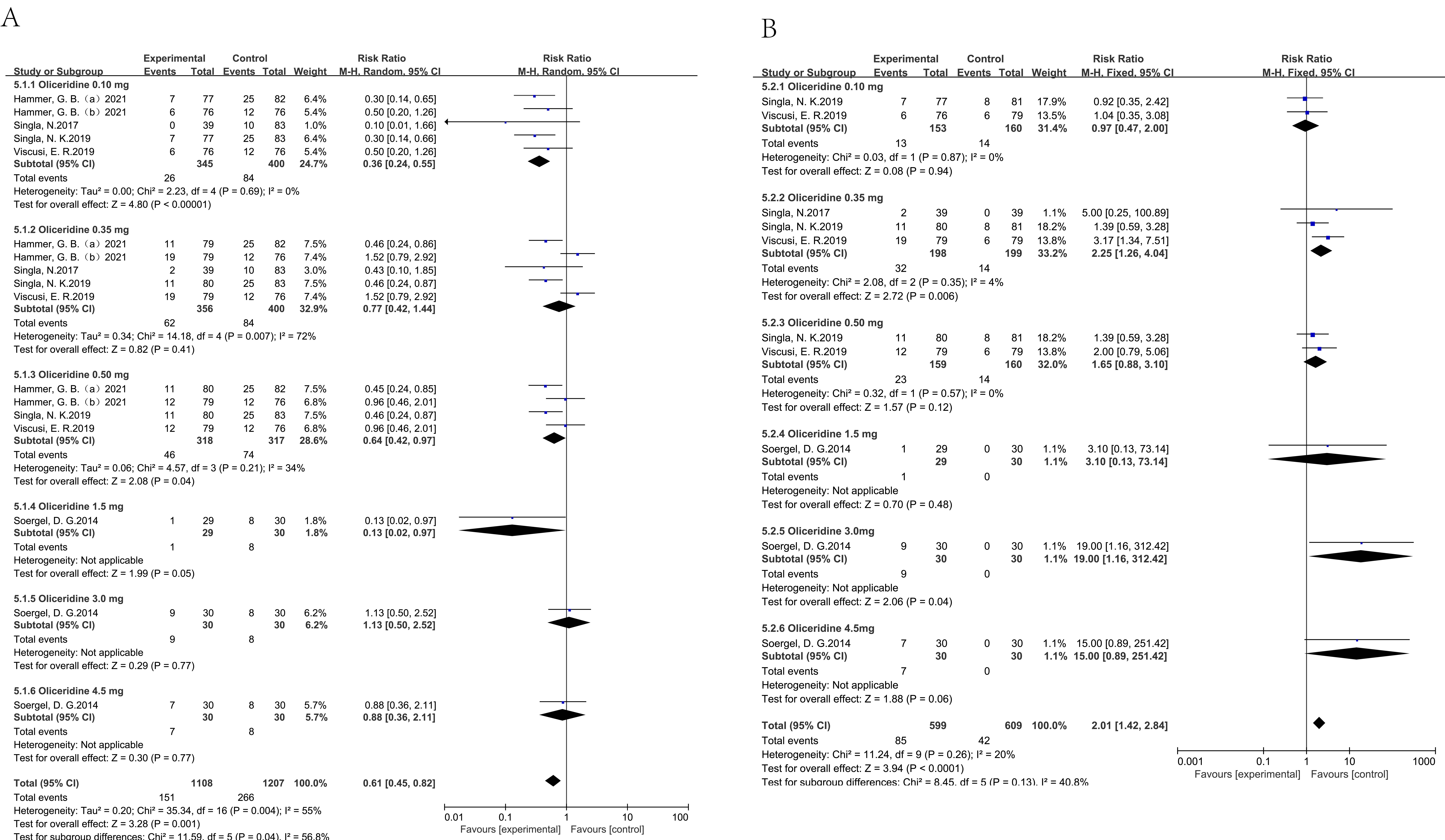

Supplement: Supplementary file 4 [file Image5.tiff]

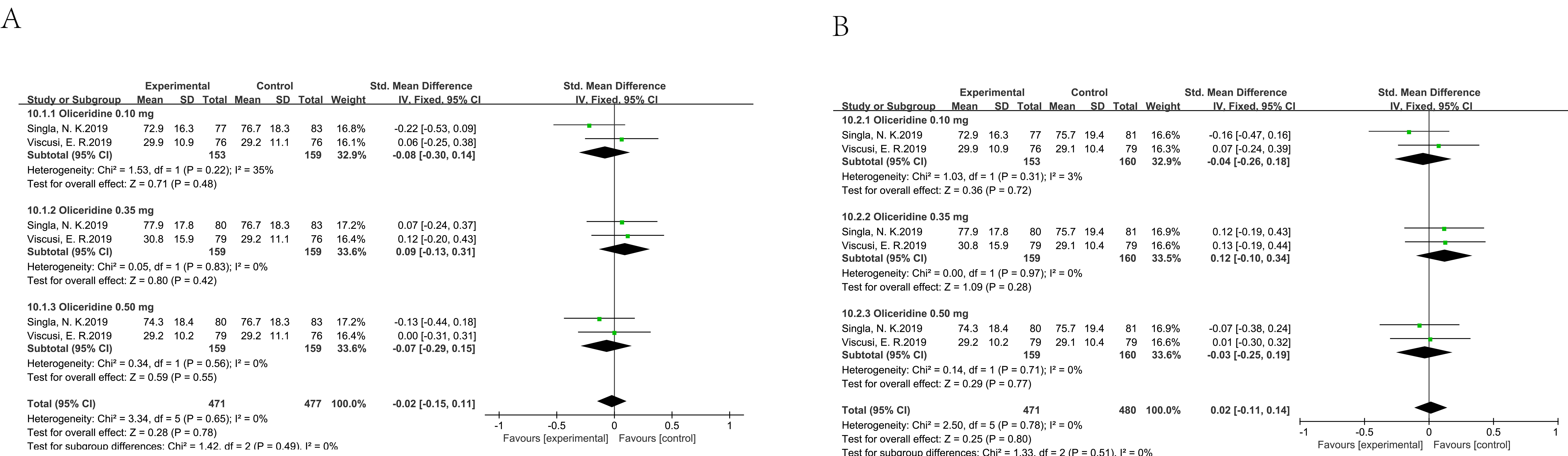

Supplement: Supplementary file 5 [file Image6.tiff]

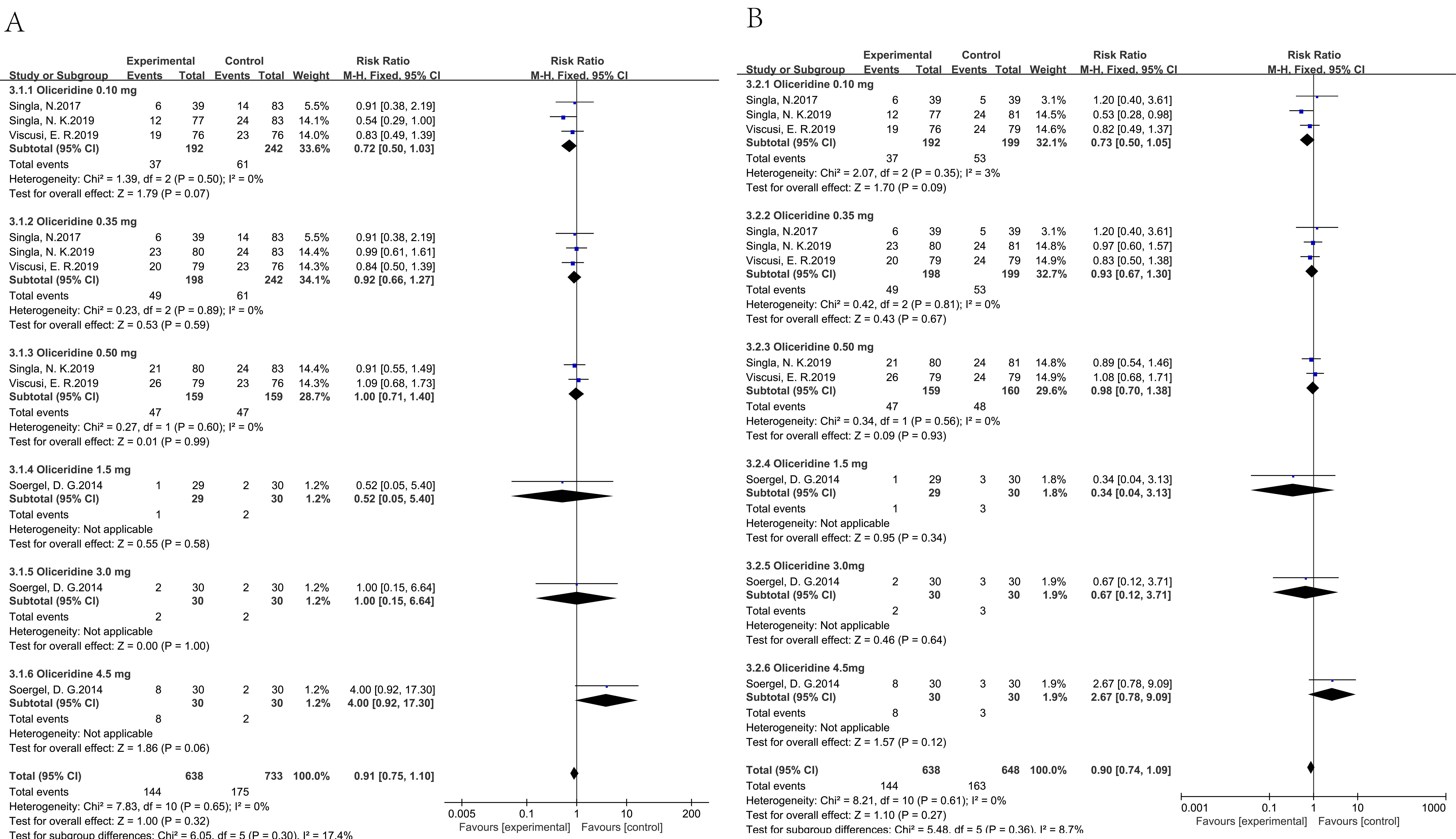

Supplement: Supplementary file 6 [file Image2.tiff]

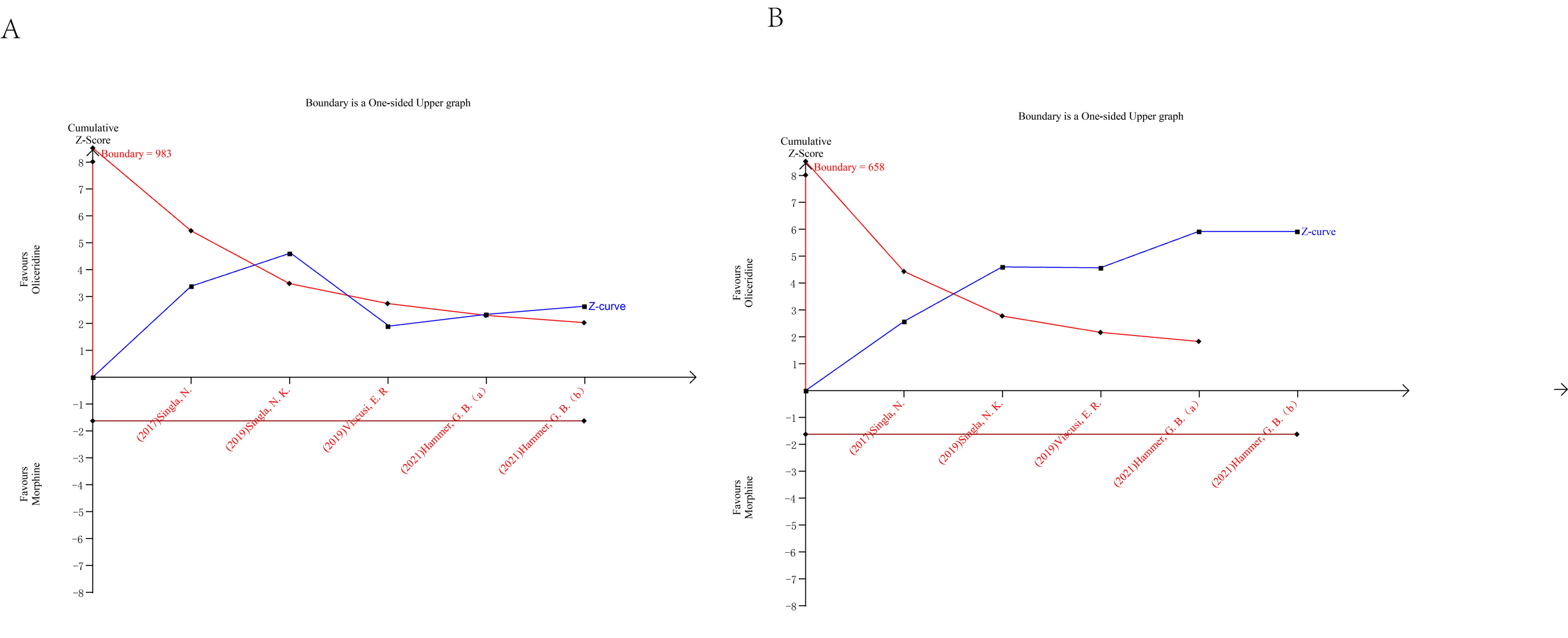

Supplement: Supplementary file 8 [file Image7.tiff]
