# Supplementary material for: Nausea and vomiting as adverse events of oliceridine: a systematic review and meta-analysis of randomized controlled trials
Source: Front Pharmacol. 2026 Apr 22;17:1779641. doi: 10.3389/fphar.2026.1779641 (PMC13144020; doi:10.3389/fphar.2026.1779641)
Supplement: Supplementary file 3 [file Table1.docx]

Supplementary Table S1. Database-specific search strategies (final run: 2024-09-30)

Database: PubMed/MEDLINE

Platform: PubMed

Coverage: Inception – September 30, 2024

Final search date: 2024-09-30

Search string (verbatim):

(oliceridine[Title/Abstract] OR TRV130[Title/Abstract] OR "TRV 130"[Title/Abstract] OR oliceridine[MeSH Terms])AND("postoperative nausea and vomiting"[Title/Abstract] OR PONV[Title/Abstract] OR nausea[Title/Abstract] OR vomiting[Title/Abstract] OR Nausea[MeSH Terms] OR Vomiting[MeSH Terms] OR "Postoperative Nausea and Vomiting"[MeSH Terms])AND

(surgery[Title/Abstract] OR postoperative[Title/Abstract] OR perioperative[Title/Abstract] OR Anesthesia[MeSH Terms])AND(randomized controlled trial[Publication Type] OR randomized[Title/Abstract] OR randomised[Title/Abstract] OR placebo[Title/Abstract] OR "double blind"[Title/Abstract])

Filters: Humans; Adults (≥18 years). Date range: inception–2024/09/30.

Limits/Filters: Humans; Adults (≥18 years)

Notes: No language restrictions; duplicates removed prior to screening

------------------------------------------------------------

Database: Embase

Platform: Ovid

Coverage: Inception–September 30, 2024

Final search date: 2024-09-30

Search string (verbatim):

('oliceridine'/exp OR oliceridine:ti,ab OR 'trv130':ti,ab OR 'trv 130':ti,ab)AND('postoperative nausea and vomiting'/exp OR 'nausea'/exp OR 'vomiting'/exp OR (ponv:ti,ab OR nausea:ti,ab OR vomiting:ti,ab))AND(surgery:ti,ab OR postoperative:ti,ab OR perioperative:ti,ab OR 'anesthesia'/exp)AND('randomized controlled trial'/exp OR random*:ti,ab OR placebo:ti,ab OR 'double blind':ti,ab)AND([adult]/lim AND [human]/lim)

Date range: database inception–2024-09-30.

Limits/Filters: [adult]/lim; [human]/lim

Notes: No language restrictions; duplicates removed prior to screening

------------------------------------------------------------

Database: Cochrane CENTRAL

Platform: Cochrane Library

Coverage: Inception – September 30, 2024

Final search date: 2024-09-30

Search string (verbatim):

(oliceridine OR TRV130 OR "TRV 130") in Title/Abstract/Keywords

AND (PONV OR "postoperative nausea and vomiting" OR nausea OR vomiting)

Limits/Filters: None beyond database defaults

Notes: No language restrictions; duplicates removed prior to screening
